# Synthetic DNA applications in information technology

**DOI:** 10.1038/s41467-021-27846-9

**Published:** 2022-01-17

**Authors:** Linda C. Meiser, Bichlien H. Nguyen, Yuan-Jyue Chen, Jeff Nivala, Karin Strauss, Luis Ceze, Robert N. Grass

**Affiliations:** 1 Institute for Chemical and Bioengineering, Department of Chemistry and Applied Biosciences, ETH Zurich, Vladimir-Prelog-Weg 1, CH-8093 Zurich, Switzerland; 2 Microsoft Research, Redmond, WA 98052 USA; 3 Paul G. Allen School of Computer Science and Engineering, University of Washington, Seattle, WA 98195 USA

**Keywords:** Information technology, DNA, Computational biology and bioinformatics

## Abstract

Synthetic DNA is a growing alternative to electronic-based technologies in fields such as data storage, product tagging, or signal processing. Its value lies in its characteristic attributes, namely Watson-Crick base pairing, array synthesis, sequencing, toehold displacement and polymerase chain reaction (PCR) capabilities. In this review, we provide an overview of the most prevalent applications of synthetic DNA that could shape the future of information technology. We emphasize the reasons why the biomolecule can be a valuable alternative for conventional electronic-based media, and give insights on where the DNA-analog technology stands with respect to its electronic counterparts.

## Abstract (web summary)

Synthetic DNA is the basis for promising technologies in data storage, barcoding, computing 62 and sercurity. In this review, the authors provide an overview of the field and its future.

## Introduction

Information technology (IT) is growing with a compound annual growth rate of 5% and is projected to continue growing at increasing rates in the near future^1^. Since the 2020 wave of remote working, cybersecurity and emerging technology solutions play an increasingly important role in the global economy^1^. So far, the chemical industry has been extensively affected by the advances of the information technology sector through the digitalization and automation of chemical processes, with the fastest growing demand being found in the pharmaceutical industry^2,3^. However, the link between information technology and the chemical industry need not be one-directional, where information technology is used to advance the chemical industry. A myriad of opportunities lies ahead when using chemistry to advance information technology, specifically to augment electronic systems with chemical systems.

To date, no molecule is more suitable for information technology applications than DNA (Table 1, Box 1). Nucleic acids possess the fundamental property of Watson–Crick base pairing, which is the basis for several DNA handling technologies, allowing for a unique scope of potential applications for computational purposes across disciplines. These handling technologies have been built on the biologically evolved properties of the DNA molecule and were originally developed for the life sciences but have since then been gradually repurposed (Box 2).

**Table 1:** DNA-based vs. chemistry-based methods for solving information technological applications.

Application	Examples of DNA methods	Examples of chemical methods
Data storage	Encoding information into synthetic DNA	Encoding information into synthetic polymers^8,95–98^, information storage using small molecules^99,100^
Barcoding	DNA product tags integrated into the material of the tagged item	Anti-counterfeiting tags^101^, chemical fingerprinting using NMR readout^102^
Random number generation	DNA synthesis for true random number generation	Crystallization robot for inorganic chemical reactions^103^
Cryptography	Information encoding in DNA strands	Authentication of valuable documents^101^, random patterns of chemicals^101^, fluorescent molecular devices^104^
Logic gates and circuits	Toehold replacement and strand migration for signal processing	Ion binding affinity to mimic AND logic gates^76^, sequential logic operation^105^, molecular keypads^106^, feedback-control loops^107^

We here review the applications of synthetic DNA that we believe have the potential to shape the future of information technology. The applications we discuss have reached different levels of maturity. Whereas some are soon to be commercially viable, others are just at stages of early research. We thus chose to discuss individual applications in the categories of data storage, barcoding, security, and computing, but realize that the different applications are at vastly different stages of development and implementation.

Box 1 Other molecular substrates besides DNA Chemical substrates besides DNA have found increasing applicability for information technological purposes. For most applications discussed in this review, there exists a chemical alternative to DNA (see Table 1). This vast field of research is beyond the scope of this review. Rather, we compare chemical (non-biomolecular) and DNA-based biochemical solutions for select IT applications and give arguments why, to date, DNA has superior properties for these purposes.

**Data storage**. Synthetic polymers are the most prevalent chemical alternative to DNA data storage. Ideally, polymers could be designed to contain a variety of different monomers, each coding for different information, thus surpassing the potential of encoding information into four nucleotides of DNA. In reality, sequentially reading the information from polymers remains challenging as automated methods are not available for synthetic polymers^8^. As long as the information contained in a single molecule cannot be read sequentially, the challenge of information retrieval does not scale well with the amount of information to be read.

**Barcodes**. Some chemical barcodes do not need to be added to the product, as the product is already composed of chemistry that can be analyzed depending on factors such as origin or geography^102^. The authentication of wine is such an example. Special nutrients such as sodium, potassium, or calcium can help determine geographical variations^108^. Another industry for chemical barcodes is anti-counterfeiting. Here, the possibility to amplify DNA tags presents a very competitive advantage, as through PCR the detection limit of a DNA sequence is a single molecule. Such a sensitivity is extremely difficult to achieve with standard chemical analytical methods.

**Random number generation**. Despite all randomness contained in chemical reactions, hardly any readout methods exist that can identify this entropy on a molecular level. Thus, such randomness cannot be easily accessed. Crystallization has been exploited to access the stochastic nature of chemical reactions^103^. However, the bit generation rate of this technology is significantly below state of the art or even DNA-based methods.

**Cryptography**. A significant part of cryptography besides encryption is product authentication. Fluorescent fibers or luminescent inks have been used for such purposes to mark valuable documents^101^. For encryption applications, molecular taggant solutions have been used to produce physical unclonable function (PUF) keys, where unique physical tags are created by adding random patterns of chemicals to a material^101^. Another recent example concerns concealed encrypted messages in fluorescent molecular devices. By adding the correct chemicals (key) to the encrypted molecular device, a fluorescent signal can be read and can thereby be decrypted^104^.

**Logic gates**. When de Silva et al. published their findings on molecular logic gates in 1993^76^, a series of research began to mimic the fundamental components of digital circuits using molecules^109^. One of the first sequential logic operations was presented by Raymo et al. in 2003 and showed integrated memory^105^. A few years later, Margulies et al. presented a molecular keypad to authorize password entries using chemical and optical signals^106^. Li et al. developed a feedback loop based on a porphyrin derivative, which was assigned different signal states depending on protonation^107^. A more in-depth review about the evolution of chemical logic gates is available in literature by Erbas-Cakmak et al.^109^.

Despite some alternatives available in chemistry, many still face challenges that have already been overcome by DNA technologies: There is currently no method to sequentially read information from synthetic polymers, and information contained in chemicals cannot be amplified to facilitate read-out. Perhaps hybrid solutions between DNA and other chemicals such as superconductive superlattices, where a 3D DNA lattice is coated with niobium (Nb) would be ideal for the future^110^. With hybrid solutions as such, the benefits of selectivity and facilitated handling, as well as the versatility of properties existing in chemistry are exploited.

**Fig. 1:**
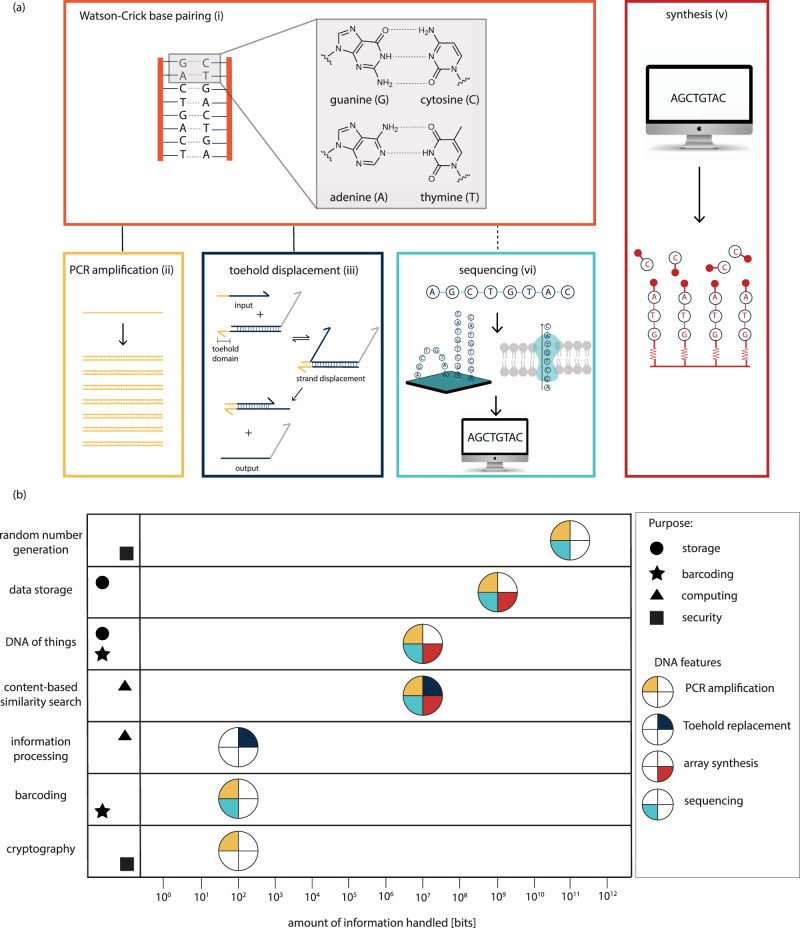
Unique DNA attributes. **a** The attributes of DNA make the biomolecule ideal for various computational applications. (i) Watson–Crick base pairing: to allow to predict the connectivity of molecules. (ii) Polymerase chain reaction (PCR) amplification: to copy a DNA molecule to an exponential multiple. PCR amplification is based on Watson–Crick base pairing. (iii) Strand displacement: to enable chemical signal processing. This attribute is also based on Watson–Crick base pairing. A single strand input molecule binds to the toehold of the double-strand gate complex and displaces one of the strands to release a single strand output. (iv) Sequencing: to read the information in DNA strands (v) Synthesis: to make new DNA molecules within minutes. **b** Applications of DNA in information technology and the amount of information described in research literature so far. The attributes of DNA (see **a**) that are dominant in the respective applications are color-coded. The purpose of each application is also shown.

Box 2 The five attributes of DNA The history of conceptualizing DNA began in 1953 when J.D. Watson and F.H.C. Crick published their seminal paper on the double-helical structure of DNA^111^. With the understanding of Watson–Crick base pairing, the following technologies for handling DNA evolved: PCR reaction amplification, toehold-mediated strand displacement, sequencing, and automated synthesis (Fig. 1).

**Watson–Crick base pairing**. *Function: Allowing binding specificity between DNA molecules*: The discovery of DNA’s helical structure led to the understanding of complementary base pairing within the DNA double helix. Complementary base pairing enables us to predict the connectivity of two DNA molecules, as DNA is composed of the four nucleotides adenine (A), thymine (T), guanine (G), and cytosine (C) of which adenine always binds to thymine and guanine always binds to cytosine. It is because of that intrinsic attribute that many applications of DNA handling (such as DNA amplification or certain DNA sequencing methods) could in fact be established. More recently, research has shown that synthetic nucleotides can be created to expand the alphabet of genetic code to eight letters in order to increase the information density potential of DNA^112^.

**Polymerase chain reaction amplification**. *Function: To copy selected DNA molecules to an exponential multiple within minutes*: The original idea of PCR was proposed by Kary Mullis in the 1980s^113^. The novelty of his work, which was later awarded with the Nobel Prize in Chemistry, was to amplify regions between two complementary strands of DNA, repeating the process over and over again, such that the product of each amplification reaction becomes part of the template pool for the next reaction thus inducing a chain reaction with exponentially increasing numbers of oligomers. When copying a DNA strand, the strand to be copied acts as a template strand. A primer (complementary to part of the template strand) attaches via complementary Watson-Crick base pairing and a polymerase (an enzyme) synthesizes the entire complementary strand of DNA. If one DNA molecule exists, millions of copies of that molecule can be made within minutes.

**Toehold-mediated strand displacement**. *Function: Enabling chemical signal processing*: The first notion of toehold displacement was suggested by Bernard Yurke et al. in 2000^114^, who built a molecular machine that was made out of three DNA strands resembling tweezers that could open and close. The mechanism was designed so that a single-stranded DNA could replace one of the strands in a double-stranded DNA complex by attaching to a so-called “toehold” domain, a single strand section of the substrate DNA. Subsequent branch migration displaces the targeted strand in the double-stranded complex^88,115,116^. Toehold-mediated strand displacement has found the most theoretical applicability in computation DNA applications that are still in their infancy of development into a stand-alone application. If the ideas mature into applications eventually, toehold displacement will most likely become more substantial to the toolbox of DNA techniques.

**Sequencing**. *Function: To sequentially read the information in individual DNA molecules*. Sequencing of DNA proceeds by reading the individual bases in order as they appear in the strand. Technology to identify individual molecules of DNA has been introduced by Sanger et al. in the late 1970s^117^ and pioneered as a sequencing method for almost 40 years. Today, so-called next-generation sequencing methods allow for higher sequencing speeds and offer much greater throughput^118–120^. These advances in sequencing technology over the past decades can be made obvious when comparing the effort that was required to complete the Human Genome Project in 2003 to the possibility of sequencing a human genome today. During the Human Genome Project, sequencing one human genome (about 3 billion DNA bases) took 13 years and USD 2.7 billion to complete. Today, it is possible to sequence an entire human genome in just one day for less than USD 1000^121,122^. State of the art sequencing technology enables the sequential reading of nucleotides in DNA strands and allows for upscaling of sequencing volumes, without added complexity.

**Automated synthesis**. *Function: Making a multitude of short new DNA molecules*: Automated oligomer (oligo) synthesis has been commercialized in the 1980s and high-throughput array-based methods have subsequently developed throughout the 1990s^21^. Automated synthesis has opened the door to a variety of applications such as synthetic gene synthesis, protein engineering, or DNA data storage^21^. In recent years, an enzyme-based alternative to state-of-the-art phosphoramidite synthesis was developed and several start-up companies were founded, using the terminal deoxynucleotidyl transferase, an enzyme that can synthesize DNA strands without any DNA template^32–34^.

## Information technology applications of DNA

Enabled by new combinations of DNA handling technologies (Fig. 1), the biomolecule has found extensive applicability in information technology systems. DNA has a high information density^4^, can be chemically preserved for thousands of years^5^, and is especially attractive for future developments due to the eternal relevance for reading DNA, given that it is the genetic material of all living organisms. Continuous advances in biotechnology and life sciences will further improve the reading and writing processes. As opposed to magnetic or silicon-based storage (consider the floppy disk, barely surviving 50 years of history), the interest in reading DNA will presumably remain for eternity. A summary of the DNA handling technologies together with the most prominent applications for synthetic DNA can be found in Fig. 1. More details about the individual handling techniques can be found in Box 2.

### DNA data storage

The desire to keep records of our lives has existed for thousands of years: cave paintings from 50,000 years ago^6^ or first forms of writing on 5300-year old clay tablets from Uruk^7^ are examples of some of the earliest records of past civilizations. Because of the storage conditions in these specific examples, it was possible for later generations to access and analyze these records to learn about our prehistoric ancestry^8^. Today, the production of digital records is growing exponentially, with the global data sphere being predicted to grow to 175 zettabytes by 2025^9^. To ensure that in hundreds or even thousands of years from now our stored information can still be accessed and read, we need durable media for information storage.

**Fig. 2:**
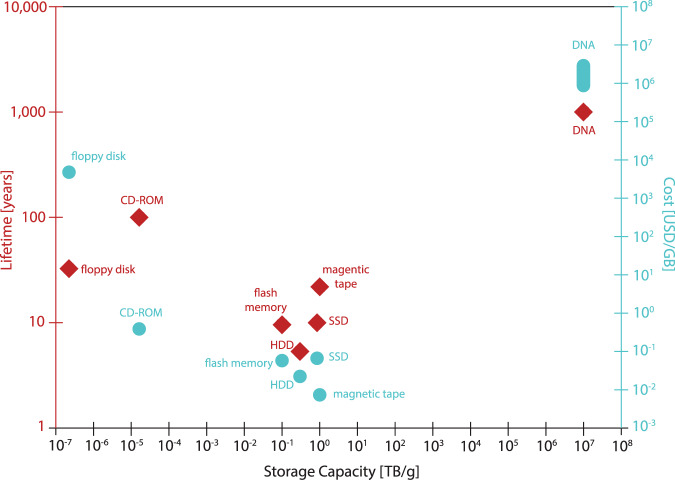
Lifetime, current storage capacity, and costs of various storage systems. Estimates for mainstream media were made with consumer pricing data. Note that (1) these costs are an estimation at the point at which this work was written and (2) that the basis for calculation is not perfectly aligned, as to date DNA data storage cannot be purchased as a routine commercial electronic storage medium and read/write infrastructure has not been scaled^10,31^.

Devices that are used for storing data today include, for example, tape, hard disks (both based on magnetic media), CD-ROMs (based on optical media), or flash drives (based on solid-state media). For long-term storage of large amounts of data, it is common to opt for storage media that offer high information density, increased longevity, as well as low energy costs at rest^10^. Of the storage media listed, tape is the most commonly used media for archival storage. However, the information density of tape is soon approaching the theoretical limit and requires copying the data stored every few decades due to inherent properties of the magnetic media. Yet, the mainstream media currently in use are soon approaching their density limits^10^, induce high-energy costs^11^, and are not made to last longer than a few decades at most, as they are prone to mechanical failure, damage due to temperature, or damage due to magnetic fields^12,13^ (Fig. 2).

A medium for data storage that overcomes these challenges, and offers a very attractive alternative to conventional media for archival information storage is DNA^14^. DNA surpasses the information density of any mainstream storage device by more than six orders of magnitude^10,14^, requires approximately eight orders of magnitude less energy at rest^10,14–18^, and can be preserved for millennia when treated accordingly^5^. Additionally, the DNA molecule is biodegradable and DNA data storage has been shown to be more environmentally sustainable than existing storage technologies^19^. The environmental friendliness of DNA can be a pivotal factor and a major reason for DNA to potentially replace magnetic tapes for archival data storage, despite higher reading/writing costs. In general, environmental impact can be classified into three different areas: (1) impact of writing data, (2) impact of storage, (3) impact of reading data. Addressing each of these points separately clearly illustrated some of the most pivotal benefits of DNA compared to magnetic tape (for archival storage): (1) in contrast to hard drives and flash drives, no heavy metals or other rare elements are required for the generation of DNA. (2) Research has shown that appropriately protected DNA can withstand centuries without the need for climate control^5^ or rewriting^8^, thereby significantly decreasing the total energy demand. (3) As DNA is biodegradable, it will cause no harm to the environment when being discarded. No heavy metals are discarded with DNA, which would be the case for other storage media. The only source of potential harm comes from dissolving the silica particles in which the DNA was preserved (to make the molecule last). This silica has to be removed using fluoride-containing solutions at low concentrations^20^. However, more environmentally friendly options are available.

**Fig. 3:**
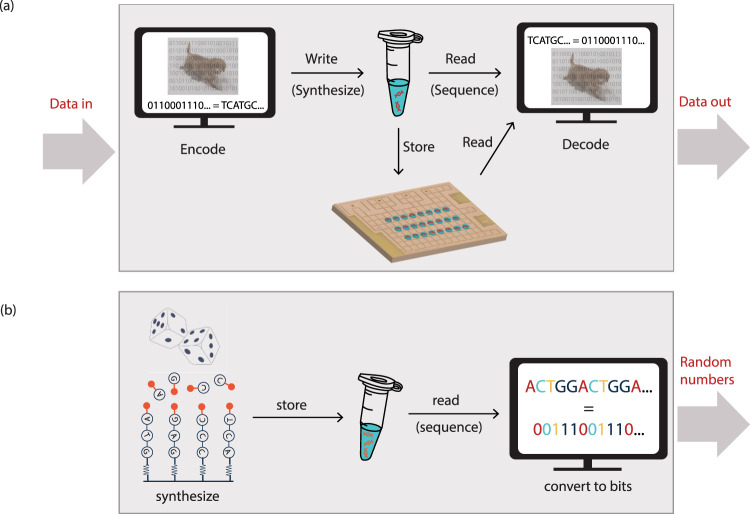
Integrating DNA synthesis and sequencing into digital information processes. **a** Storing information in DNA. The bits composing a digital file are translated to DNA nucleotides, which can then be synthesized using array-based synthesis technology and stored. To retrieve the file, DNA is amplified using PCR and sequenced. **b** Process of random number generation. DNA strands are synthesized with nucleotides being added randomly to the growing strands. The random number can then be physically stored in a tube containing the DNA and subsequently read by sequencing technologies. Post-processing steps ensure that the synthesized number is truly random when converted into bits.

The process of DNA data storage is illustrated in Fig. 3a and proceeds as follows: A computer maps a string of bits (zeros and ones coding for a digital file) to sequences of DNA (for example with a system such that 00 = A, 11 = T, 01 = G, 10 = C) using so-called error-correction codes. This also introduces redundancy, so that if information gets lost during the storage process, the digital file can still be retrieved and read at later stages, as well as an index to differentiate between the individual sequences. By means of automated synthesis, millions of copies of these DNA strands are then physically generated^21^. The DNA strands can be stored, which is commonly done by freezing the DNA in solution, drying the DNA, or encapsulating the DNA molecules in small silica particles^5,20^ to shield the stored information from environmental factors. It is not necessary to store millions of copies of each DNA strand. While theoretically 455 EB^4^ can be stored per gram of DNA, technically, it has been shown to be possible to fully recover the digital file when 10 copies of each DNA strand are present^22^. This allows for an extremely high information density of 17 EB/g^22^. To retrieve the digital file again, the DNA is amplified using PCR to regenerate millions of copies of the DNA strands, which can then be read using sequencing methods. Subsequently, using error correction, the DNA sequences can be decoded back into the strings of bits, which make up the digital file^4,5,22–26^. To date, files of sizes up to 200 MB have been stored in DNA^24–26^, and calculations show that in theory, all information produced globally in one year could be stored in 4 g of DNA^8,13,17,27^.

There are a few major technical challenges that DNA as a medium to store data will have to overcome. The discrepancies in terms of throughput of data writing and reading^10^ and the cost between the tape storage and DNA storage are enormous. For DNA storage to be competitive with magnetic storage, it would need a throughput of gigabytes per second. To date, the throughput of writing DNA is estimated to be in the order of kilobytes per second. Additionally, there is still a large discrepancy between the time required for writing and reading data stored in DNA compared to data stored in magnetic storage alternatives. Besides intrinsic storage features of reading and writing data, random access (the ability to choose a specific data item out of a larger data set)^10^ in DNA data storage cannot proceed as we know it from conventional storage methods, as there is no physical organization between the data items contained in a larger pool of DNA strands^10^. It has been shown that different primers corresponding to different data items, as well as magnetic beads extraction can be used to implement random access to DNA data storage^24,28,29^. Rewritability of information is another major challenge for DNA data storage, as data is stored in a read-only format^8^. Yazdi et al.^29^ presented concepts of DNA rewriting by two DNA editing techniques. The first, gBlock, was used to combine a new strand (containing new information that should replace old information) with an old strand (containing old information that should be replaced by new information) using PCR, thus rendering the edited strand (a combination of the new strand and the old strand). The second method for rewriting information is the overlap-extension-PCR (OE-PCR) method, where new information is introduced via an overhang on a primer that can be added to the file using PCR^29,30^. However, both procedures are not very practical as significant amounts of DNA are required per rewriting step and parallelization of the process for the scalable application currently remains unsolved.

Although DNA data storage is seen as one of the most advanced and integrated technologies using DNA, depending on literature reference or supplier, one megabyte of encoded information still costs around USD 800–5000 USD/MB^10,31^. To make DNA data storage compete with state-of-the-art archival tape technology, costs of writing would have to be reduced by up to eight orders of magnitude, and costs of reading would have to be reduced by five orders of magnitude. Additionally, speeds of writing would have to increase by six orders of magnitude, whereas speeds of reading require an improvement of up to three orders of magnitude. Novel DNA synthesis techniques have developed in recent years and present a very interesting alternative to state-of-the-art phosphoramidite coupling chemistry. Such a new technique is enzyme-based synthesis, and several start-up companies have already been founded in the field, making use of an enzyme that can synthesize DNA strands without any DNA template^32–34^. There is hope that synthesis with enzymes can reduce associated costs by the required factor. More in-depth reviews of DNA data storage, also considering the gaps between theory and applications, are available in literature by Ceze et al.^10^ or by Rutten et al.^8^.

An initial example of DNA data storage has already been demonstrated using visual art. Artists together with researchers have come up with ideas to incorporate the idea of DNA data storage very symbolically: At the University of Washington, a visage of Rosalind Franklin on a background of 2000 images submitted by the general public was painted and hung up to commemorate the biochemist and pioneer in understanding the molecular structure of DNA. What is special about this piece of art is that the 2000 digital images were encoded into DNA and the DNA was mixed with paint such that when scraping off a part of the paint, all images can be retrieved and viewed digitally using standard DNA data storage procedures^35^. Very similarly, researchers from ETH in Zurich and the Technical University of Munich have stored the album Mezzanine from the British band Massive Attack in DNA. They encoded the digital album into a sequence of nucleotides, which they synthesized to obtain the music album physically in form of DNA. The DNA was encapsulated in silica particles for mechanical stability, and then mixed into the spray paint so that any artwork created with this spray can contain the file of the music album^36,37^. These artworks give a very unique signature to the paintings, which will remain accessible for millennia.

### Barcoding and product tagging

A variety of product tagging forms exist in our society. Well-known are universal product code (UPC) barcoding for tracking items in stores, radio-frequency identification (RFID) for tracking inventory goods, or quick response (QR) codes for accessing information with a mobile phone. However, in some cases, these conventional tagging methods are inconvenient or even impossible to implement, for example, when the object changes its physical state during its lifetime, when the object is small, or when the barcode should remain unseen. This could, for example, be the case with pharmaceuticals (i.e., small tablets), food items or objects containing secret messages that are not to be visible to the human eye. One commercial example, where such unseen barcodes are attractive is the tracing of textiles in order to make supply chains more transparent. The company named Haelixa is active in this market and mixes DNA strands with cotton to provide users with a reliable tool to trace and identify their products throughout the entire supply chain. This promises increased sustainability and integrity of textiles. For such applications, molecular barcodes offer a valuable solution^38^.

It is important to note the distinction between uses of the term DNA barcoding, as DNA barcoding is used for classifying species (different gene regions function as barcodes to identify organisms)^39,40^, to study molecular systems (for example in the form of unique molecular identifiers to eliminate PCR bias)^41^, or to tag products^42–44^. A molecular product tag is a pre-defined amount of DNA that is added to the building blocks of a certain substance. Thus, the material of the substance contains the barcode once assembled.

Molecular product tags must fulfill certain requirements to be suitable for their application. Besides the need to remain intact throughout the product lifetime (i.e. such that external factors do not damage the barcode), the tags must be non-toxic (i.e. if the product is to be digested). Additionally, the limit of detection, as well as implementation costs, have to be considered. DNA is a biomolecule that degrades when exposed to harsh environmental conditions such as elevated temperatures, moisture, or UV light^45^. This problem can be circumvented by encapsulating the DNA^5,46,47^. As DNA and many encapsulants are non-toxic materials and the limit of detection can be as low as 0.001 ppm, which is several orders of magnitude lower than with chemical alternative taggants^48^, making DNA extremely suitable for product barcoding in these terms. Lastly, the associated cost depends highly on the synthesis and sequencing procedures required for reading and writing the DNA barcodes as well as the scale of barcoding used. In general, if the value added to a product by tagging it is higher than the associated costs of the process of integrating DNA barcodes into the product, then the DNA barcode proves to be particularly suitable^48^.

The simplest form of a DNA product tag would be the presence or absence of a synthetic DNA strand, which enables binary classification of objects (i.e. if the drug is an original, the DNA sequence is present in the material, if it is a counterfeit, the DNA sequence is not present in the material)^49^. Using PCR it is possible to determine the presence of such a product tag. DNA barcodes can become infinitely richer in data by encoding more information into the DNA sequence. The content of this information can be as short as a few bits coding for certain parameters, or can be large as a document, several megabytes in size^43^. To read out the information from information-rich barcodes, sequencing of the DNA is required. Sequencing reads can then be analyzed, and the information decoded from the readout data. More recent techniques for barcode readout have focused on raw signal processing of sequencing runs^44^. This is possible if the DNA barcode is designed in a way so that the raw signal of one barcode molecule is significantly different from the raw signal of another barcode, and has been shown using the low-cost MinION from Oxford Nanopore Technologies^44,50^. A recent DNA-based tagging system called Porcupine shows that rapid writing and on-demand readout using raw signal processing is possible, lowering costs compared to previous tagging solutions^44^. This offers readout times of just 1–3 min, which promises to be competitive for applications in low-resource environments or commercialized product tagging^44^.

DNA tagging has found great interest in industrial applications to minimize counterfeit products. Several industries have adapted DNA barcoding methods for product control and supply chain accountability, such as Eurofins offering extended leather traceability by integrating DNA tags during leather production steps^51^, or Haelixa, offering proof of authenticity for textiles, emeralds, or gold^38^, but also the food industry to fingerprint wine, helping verify grape identities^52^, to fingerprint ginseng or to help distinguish between important herbs in Chinese medicine^52^. These examples show that DNA product tagging out of all applications has already been established commercially. For anti-counterfeit applications, although very well hidden in the products, DNA product tags could, like most other commercial tags, be exploited. For this the DNA sequence would have to be known and reproduced by the intruder, requiring extensive laboratory sequencing and DNA synthesis efforts.

Recently, Berk et al. presented a method for reading out DNA security tags, by the human eye or a smartphone. This allows for minimal technology and user training required. The researchers based their new technology on toehold-mediated strand displacement reactions to detect the presence of certain oligos in the taggant. When these oligos are present, a fluorescent signal emerges that can be detected using a flashlight. The simplicity of such methods will likely be attractive for many future applications of barcoding, as the ease of end-user applicability significantly increases the market size of DNA product tags^53^. The opportunity behind barcodes and product tags can, for example, be estimated by looking at the market size of inventory tags, which amounts to 4.6 billion USD in 2020 and is expected to grow to 6.2 B USD in 2028.

**Fig. 4:**
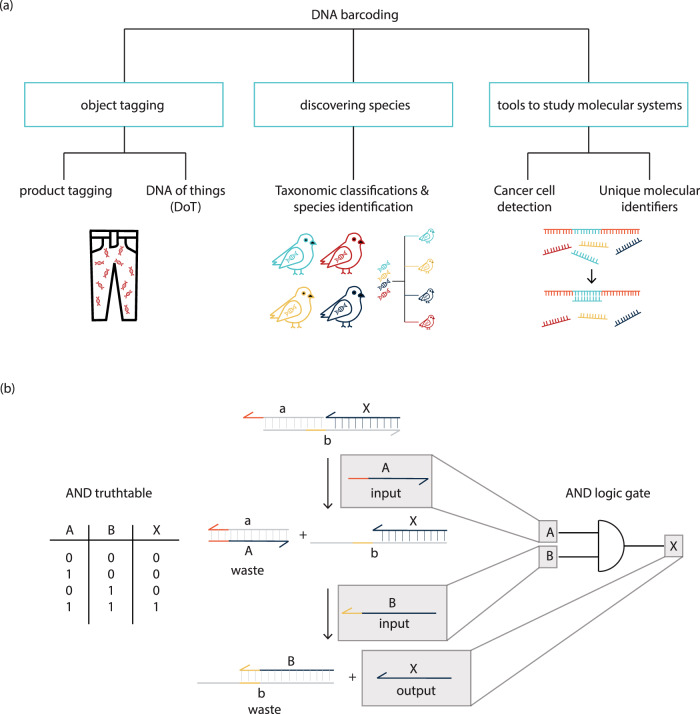
DNA for identification and computing applications. **a** Applications of DNA barcoding. Applications include product tagging and DNA of things, as discussed in this review, but also species identification, cancer cell detection, or unique molecular identifiers, which lie outside of the scope of this review. **b** AND logic gate and truth table. DNA logic gates with input signal strands «A» and «B» and output signal strand «X». Toehold regions are marked in orange and yellow, signal strands are marked with capital letters and complementary strands are marked with lower case letters. Signal strands are dark blue whereas all other strands are gray. Input signal A binds to gate complex (containing strands a, b, and X) and displaces a part of strand b to form “waste” complex A-a. Input signal B is then added. It displaces output signal X from the complex to bind with b, forming “waste” complex B-b. Single strand X is the output signal of the gate.

In summary, DNA product tags are sequences of DNA that are added to a product for identification or traceability applications. The DNA barcodes discussed in this work are particularly relevant for the tracing of commodity products and some commercial services already exist, using DNA as proof of authenticity. However, the spectrum of applications DNA barcodes is much larger than just the product-tagging industry (Fig. 4a). As mentioned above, DNA barcodes are used for taxonomic classification, to identify species from a reference database by amplifying the target DNA barcode region using PCR, but also for the detection of rare tumor cells in the human body^54^, to eliminate PCR bias in the form of unique molecular identifiers^41^, or as a DNA of things storage architecture (which will be discussed in the subsequent section)^43^.

### DNA of things

The idea of DNA of things (DoT), derived from the term “internet of things” (IoT), was first introduced in 2020 with applications ranging from information storage to DNA barcoding of objects^43^. The concept is based on 3D-printing of objects with memory, by encoding the desired information in DNA, encapsulating the DNA in silica particles and fusing the DNA-containing particles into various materials which can then be used for 3D-printing. Thus, a material could, for example, contain digital information about its identity in the form of DNA—a concept parallel to genomic DNA coding for each person’s identity in the human body^43^.

The procedure, similarly to the DNA data storage procedure, starts with an error-correction code converting a digital file (bits) into DNA (nucleotides)^25^ and adding information redundancy, such that a given dropout of sequences is allowed for a full recovery of the digital file. The DNA strands are then synthesized and encapsulated in silica particles to mitigate stress and decomposition of DNA at elevated temperatures and to enable the miscibility of DNA with the matrix material of the final object. This encapsulation step can be seen as synthesizing a thin glass layer around DNA molecules. Applicable materials for DNA integration and subsequent 3D-forming are for example polycaprolactone (PCL) a biodegradable polyester, or Plexiglas. When 3D-printed, the object will contain the DNA information encoded. However, as the silica layer is just about 15 nm thick^20^, the beads cannot be seen within the 3D-printed object (even if the object is transparent like Plexiglas). The information can be retrieved from the object at any time by cutting off a part of the 3D-printed object (partially damaging the object) and dissolving the material to isolate the beads containing the DNA. The DNA can then be accessed from the beads using publicly available procedures^20^, and, using PCR, the strands coding for the digital file can be amplified. Using state of the art sequencing technologies, the file can then be read and decoded back to the original digital file^43^.

Presented as a proof of concept, DNA of things finds itself as a variant of both DNA data storage and DNA barcoding. It presents a niche application of data storage and barcoding combined and potential future applications for the DoT-technology may include labeling of pharmaceutical products, building materials, or electronics for which product control is required at levels that cannot yet be achieved with conventional tags. The particles containing DNA are considered non-toxic, potentially allowing for special medical applications or dental implants. As also shown, the DNA can also be stored in materials to allow the transfer of hidden messages or secret information, invisible to the human eye^43^. In summary, DNA of things combines the DNA data storage technology and the idea of barcoding to add information to the object that cannot be seen with the human eye. Comparable to DNA data storage, DNA DoT can be synthesized by commercial manufacturers. Once the information is to be retrieved from the 3D-printed object, PCR amplification can be used to amplify the desired sequences to provide enough copies for sequencing the file. The beauty of PCR for this application is shown in the minimal amount of sample required. Sequencing the DNA file gives a digital list of all DNA sequences present in the pool. Subsequent error correction decodes the DNA strands back into the encoded digital file on a computer.

### Random number generation

As an application, which is of great interest to security schemes, random numbers are particularly important to society today, as people store and access more information in the cloud, requiring increasing cybersecurity^1^. Especially since the shift towards remote working during the Covid-19 pandemic starting in 2020, network security systems, as well as encryption of exchange information, are becoming essential^55–58^. Random numbers are tools for encryption and protection against undesired interference of transferred information. Today, state-of-the-art random number generators provide a throughput of about 500 MB/s^59^. Two types of random number generators can be distinguished: true RNG (where features of physical processes are converted to random numbers)^60,61^, and pseudo RNG (where algorithms produce random numbers)^60,62^.

A potentially competitive source for true random number generation is automated DNA synthesis, which enables a true random number output of 0.3 MB/s^63^. DNA offers a great independent source of entropy that is air-gapped and orthogonal to other RNG sources. This entropy can be used directly or as a secure seed into a pseudo-RNG. The mechanism for DNA random number generation (Fig. 3b) starts with the mixing of the four different nucleotides, before these enter the chamber for solid-state DNA synthesis and form DNA strands with no perceptible order of nucleotides. One synthesis run can produce more than 7 million GB of randomness (as depicted in Fig. 1b). By means of PCR, the DNA strands can be amplified, then sequenced, and converted to bits.

Automated synthesis allows for millions of different DNA strands that are generated within a few hours. The DNA strands can then be read using state-of-the-art sequencing techniques. The sequencing process can be scaled up or down as desired to accommodate for various sizes of random numbers synthesized and results in a digital file of DNA nucleotides, which can then be encoded into bits. By encapsulating the DNA using silica particles^5,64^, is it also possible to keep the random number in a physical form, completely air-gapped, as a source of entropy that can be stable for millennia.

The bottleneck for this technology, unlike for DNA data storage, is DNA sequencing and, although this process is completely automated, the costs and throughput still limit the capabilities of producing large volumes of random numbers by using DNA. Despite sequencing latency, DNA random number generation offers great advantages: the large volume of DNA strands (random nucleotides) synthesized inexpensively in a short period of time allows for a great source of random numbers. With the latency of currently applied DNA sequencing technologies (minutes to hours), random number generation through DNA can currently not compete with other physical-based true RNG generation techniques. However, as writing and reading the random numbers are two modular approaches, further improvements of sequencing technologies and the envisioned omnipresence of DNA sequencers, may allow DNA random number generation to become attractive in select applications. Additionally to improving the cost and throughput aspects of sequencing, an overall automated process for obtaining random numbers from DNA synthesis would be required, similar to the one proposed by Bogard et al.^65^.

### Cryptography

Cryptography, in its modern sense, involves mathematical disciplines to develop techniques securing digital data systems as well as the transmission of information against adversarial attacks^66,67^. The evolution of cryptography can be categorized in two different eras: Before 1980, when cryptography was mostly art, used mainly by governments and military organizations^67^, and since the 1980s, when cryptography developed into a science, eventually used by everybody in the form of passwords, credit card transactions or the internet^67^. The correct and secure implementation of cryptography will be vital for the growing volumes of transactions made through the internet^68^. The principle of exchanging cryptographic information is the following: Parties A and B want to share a secret message. Party A encodes the plain-text message, and both, parties A and B must have access to a key (also called a one-time pad) to decode the message. For complete security, this key has to be random, remain completely secret at all times, and must never be re-used.

The idea of designing a cryptosystem made of DNA molecules was first introduced by Gehani et al. in 1999^69^, who proposed techniques that were in principle unbreakable. In the same year, Clelland et al. created the first DNA-based steganography scheme to conceal messages within a large pool of random DNA^70^. Their idea was to encode a plain-text message into the four nucleotides of DNA, subsequently mixing the message-DNA with human genome DNA for the message-DNA to be hidden. With knowledge of the primer to amplify the message-DNA as well as the encryption key, the recipient could then use PCR to decipher the DNA message.

Mainly inspired by the high information density of DNA, various data encryption schemes, exploiting the benefits of Watson–Crick complementary base pairing was developed thereafter. Some of the methods for decryption relied on PCR or gel-electrophoresis so that there was no need for sequencing^71^, which seemed advantageous at the time when sequencing readout was slow and more costly than it is today. The paradigm shift of DNA cryptographic thinking began when researchers not only made use of the sequence information in DNA strands but started taking advantage of the high structural versatility DNA has to offer^72^. This enabled methods for secure communication like DNA origami cryptography (DOC), generating scaffold DNA nanostructures that allow for complex patterning of molecules. This scaffolding technique opens up an enormous design space, allowing for a theoretical key size of 700 bits (in contrast to the advanced encryption standard, which uses 256 bits^73^). The DOC architecture also allows for differential access to only parts of the encrypted message and overall guarantees confidentiality, integrity, and availability (CIA) of information opening the door for biomolecular next-generation information security^72^. Despite these vast opportunities, intrinsic limitations of characterization methods restrict usage of the hypothetically huge keyspace (as resolution or 3D characterization methods limit the level of detection), and in addition, long read-out times of a few hours cannot yet compare to electronic computation standards^72^. More in-depth reviews of DNA as a tool for cryptography are available in literature by Lustgarten et al.^74^ and by Zhang et al.^75^.

### Information processing using DNA circuits and DNA neural networks

Information processing involves the input of a signal in one form, transforming it to another form. We will focus here on information processing through logic gates as well as through neural networks, as these systems have been adopted by researchers to construct a DNA-based analogy.

Logic gates are foundational to silicon-based electronics and modern-day computers and require binary inputs to produce a single binary output. An example of an AND gate can be seen in Fig. 4b. The corresponding truth table shows how the combinations of input signals (A and B) lead to an output signal (X), where a 0 represents the absence of an input/output and a 1 represents the presence of an input/output. Neural networks are inspired by natural brains and are designed to transmit information similarly to a neuron, where a signal is passed across synapses.

The first idea of a molecular logic gate was introduced in 1993, by de Silva et al.^76^, who demonstrated signal processing using the binding affinity of ions. Depending on which ions bound, a fluorescence signal could be observed. The same logic for signal processing can be obtained with DNA molecules where DNA sequences function as nucleic acid binding domains. DNA offers the advantage that due to its specificity of Watson–Crick base pairing, it can code for an enormous variety of signal inputs. On this basis, research has been conducted to develop biomolecular switches using enzyme catalysis^77–82^, and biomolecular switches are driven without enzyme catalysis. The former makes use of (catalytic) nucleic acid domains for selective binding to targets in order to signal their presence^78^. The latter has become of particular interest for the development of DNA-based signaling cascades and logic gates^79,83,84^. DNA-based logic uses short oligonucleotides as input and output signals. An example of a molecular AND gate can be seen in Fig. 4b. Initially, a dsDNA molecule (gate complex) is present in the pool. Upon the addition of a single-strand input molecule (strand A), the computation step begins and input A can bind to the toehold of the gate (marked in orange) to form an inert “waste” complex with the complementary strand (a). A second input (strand B) is then added to bind to the second toehold (yellow). Signal B displaces output strand X by binding to its complementary strand b to form another inert waste complex. Output signal X is left as a single strand. Verifying this mechanism with the truth table: In the absence of either input A or B, no output X will be released. In the presence of both, input A and input B, output signal X will be released. An output signal can either function as an input strand for another computation further downstream, or it can release a fluorescent signal for readout. Input logic (“0” or “1”) is controlled through concentration adjustments of the input oligonucleotides^84,85^, and gate function is only determined by base pairing and breaking. As the molecular morphology of the input and output signal of DNA logic gates is the same, cascades of signals can be built to obtain multilayer circuits^84^. Using this motif, large DNA circuits have been constructed containing up to 130 DNA strands^85^.

Besides Boolean logic, other processing approaches have been shown to transmit DNA signals. For example, neural networks^86^. A neural network takes inputs and processes them to give an output. The neural network itself consists of many small units, called neurons that are stacked in different layers. Neurons of one layer are connected to neurons of the next layer by weighted connections so that signals are transmitted through all layers of the neural network to give an output. Cherry et al.^87^ have constructed a neural network of DNA signaling that recognizes a pattern by comparing it to patterns in its memory to identify the pattern to which it is most similar. The functions of the network are broken down into individual chemical reactions, in which the outcome is determined by the concentration of the individual input signals, and readout is obtained through fluorescence. In summary, DNA information processing is dependent on automated synthesis, Watson–Crick base pairing as well as toehold replacement (Fig. 1). However, the success in building large DNA circuits is mainly attributed to the principle of toehold-mediated strand displacement, the modular construction approach, and digital logic. During signal processing, because of the toehold that serves as a region for an incoming strand to attach to, an input signal strand can induce strand migration, resulting in the originally bound strand to be released from the complex. Binding to the toehold and the substrate strand is possible because of Watson–Crick base pairing. The kinetics of hybridization of an incoming strand can be tuned by adjusting the binding strength and length of the toehold due to the predictability of binding strengths between complementary bases^88^.

It is relevant to note that this field of computational research is further away from any specific application in information technology, however, its application in biology is clearer, including DNA-based imaging probes, prototypes of smart therapeutics as well as drug delivery systems^89^. Although the concepts discussed in this work are of great interest to many in the signal processing industry, it is not yet clear where this technology can find applicability in information technology for future implementation.

### Content-based similarity search

State-of-the-art content-based search algorithms exist for either exact search or for content-based similarity search. The difference between the applicability of the two usually lies within the complexity of the data. Exact search, as offered by, for example, databases or some search engines, is mostly limited to text search. Similarity search comes into play when data is noisy and complex, when the difference (or similarity) between data items can often be found only with abstractions from bit-level to higher-level representations (e.g., features in machine learning systems). An example could be two separate recordings of a person saying the same word.

Data search in an archive or database will become important for DNA storage if conventional storage methods become obsolete. However, most research of DNA data readout so far has focused on retrieving data items by unique identifiers, but not by their actual content^90^. Recent works presented a solution to how content-based similarity search using data encoded in DNA could shape future systems. The researchers used algorithms for learning the mapping from image features to DNA sequences so that similar images were mapped to DNA sequences that are more likely to hybridize. Visually similar images could then be retrieved from the pool of images using a query images by means of DNA hybridization. As the DNA was designed so that visually neighboring images would be represented by similar DNA, the complementary DNA strand of the query image could bind to regions of DNA coding for similar images. For example, consider images containing different kinds of animals. Similar images (i.e. all images containing birds) can then be retrieved from the pool of DNA as the DNA strand coding for a bird image is similar. However, the DNA strand coding for a cat image would be significantly different. If a query DNA strand to retrieve bird images would then be used to retrieve images, DNA coding for cat images would not bind to the query DNA strand, as the two strands will not hybridize. If there is a match between the query strand feature and the image feature, hybridization between the two DNA strands would be possible. The researchers demonstrated features set retrieval out of a pool containing 1.6 million feature sets^90^. A potential limitation of this content-based similarity search system is that the feature extractor for a particular database remains the same throughout the databases’ existence. If the database grows with time and with that growth the database changes its statistical characteristics, the feature extractor may not be optimal for the expanded set of images. As such, reindexing may be needed if the database evolves too far from its initial statistical distribution or if a new feature extractor is desired. However, further development of DNA-based similarity search could greatly benefit the DNA data storage world as well as existing electronic systems, leading the way towards hybrid electronic–molecular computation devices^90^.

In summary, automated synthesis is required for writing large pools of DNA, so that all the features that were previously mapped from images to DNA can be physically handled. Watson–Crick complementary base pairing, as well as PCR amplification, are required for retrieving query images, as hybridization between query and target features is the key to successfully finding images with similar content. High-throughput sequencing is then used to read the sequence of the features in the image retrieved and to decode that sequence back to the digital file. Content-based similarity search is still in its infancy, and while it has shown promising prototypes, making this system practical requires building a fully automated end-to-end DNA electronic hybrid system.

## The future of DNA information technology

**Fig. 5:**
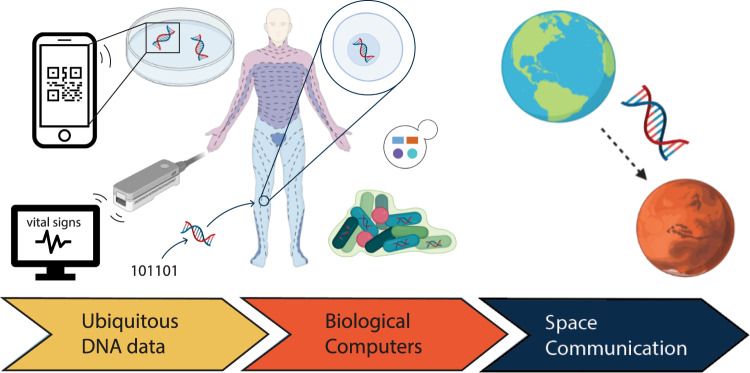
The future of Information Technology in DNA. Speculative perspective on how synthetic DNA could be integrated into society and technology in the near and far future. Soon, DNA reading and writing technologies could become everyday tools, eventually becoming more integrated into health monitoring and bio-sensing. Biocomputers could record bioprocesses from the human body within cells or bacteria. Further along in time, DNA could one day be used as a tool for communication between extraterrestrial settlements (not further discussed in this work). Although these events are illustrated in a linear fashion, components of each speculative idea could, of course, come to life on a very different timescale or in very different environments than predicted in this figure. Created with Biorender.com.

How will DNA applications be relevant for information technology in the future and which areas of research will become prominent for applications of biomolecules? Although it is only possible to speculate about how the future will unfold, current research trends can already give us an indication of where DNA and information technology could be headed in the near and far future (Fig. 5). Recently, large players in the field have founded the DNA Data Storage Alliance with the mission to promote DNA as a storage system, and have already attracted almost 50 member organizations. With attention and investment flowing into this research field, processes that to date are too expensive for making DNA data storage viable for mainstream adoption may quickly evolve to make reading and writing technologies everyday tools. In 2013, Goldman et al predicted that if costs of DNA synthesis decreased 10 times, DNA would be a cheaper option than tape for archives of a few MB and storage horizons in the order of 50-500 years (assuming tape is re-written every 10 years)^23^. However, it is difficult to predict when the prices of synthesizing DNA will drop. While advancements have been made in literature reports on lower-cost synthesis techniques^31,34,91^, to date commercial DNA synthesis costs have not drastically changed^26^. Cheaper and faster ways to synthesize and sequence DNA will facilitate progress in applications not only limited to DNA data storage, but also including DNA barcoding, DNA of things, or random number generation. However, these applications are industrially not as pronounced as DNA data storage and make predictions of future commercialization more difficult.

Besides the cost of DNA synthesis and sequencing, a major bottleneck includes slow commercialization efforts to take research findings outside of the laboratory and into applications that can be accessed and utilized by anyone. Only then would end-users be able to use DNA for everyday IT processes. An example could be reading DNA barcodes by using a smartphone. The proof-of-concept is still in its very early research phase, however, ideas like this would help to alleviate the requirement of any laboratory equipment^53^.

Another growing area is people’s interest in health monitoring and biosensors. Reaching much further into the future than just the next few decades, the use of DNA could eventually present a complete paradigm shift—from smartwatches recording a limited set of people’s vital signs to DNA recording all bioprocesses of our bodies, molecularly sensing everything that happens in our cells and organs^92^. Immunological memory systems using CRISPR-Cas have already been demonstrated to store foreign DNA (e.g., from viral infections) in genomic arrays in the form of short sequences^93,94^. Similarly, engineered bacteria have been shown to sense extracellular and intracellular signals in order to monitor human health^92^. Perhaps eventually such applications will not only offer personal diagnostics but also help uncover unknown and novel biological pathways in the body.

We can speculate further about the integration of DNA into information technology, ultimately only limited by our own imagination. However, one important question is already eminent and will remain important in the future: Just because we can, is it ethically responsible to further program biology? If engineers of the future enable molecular monitoring to record all vital signs of the human body in DNA, could these applications and tools be used to harm others? What is our responsibility as scientists and engineers, when shaping the future with novel and controversial new technologies? A careful balancing of opportunities and risks will be of great importance, especially if information technology is to be used to change biology. Instead of focusing on biology itself, this review describes how advances in biology and chemistry surrounding the DNA molecule can be used to advance information technology. Further research in this field will eventually enable new ways of generating, transmitting, calculating, storing, and reading digital information.

### Reporting summary

Further information on research design is available in the Nature Research Reporting Summary linked to this article.

## Supplementary information

Reporting Summary
